# Dental Pathology and Surgery

**Published:** 1872-12

**Authors:** J. C. Ross


### Dental Pathology and Surgery.

BY DR. J. C. ROSS.

*Read Before the Tennessee State Dental Society.*

Pathology has been defined *diseased physiology, and physiology of disease.* The first relates to diseases in common; the second considers the particular history of each. It is subdivided into internal and external, or medical and surgical.

In your committees report it will be quite sufficient that a few thoughts be suggested in regard to dental pathology, and the external or surgical treatment relating thereto.

It is not the purpose here to enter largely into the subject, which indeed is almost interminable and exhaustless, but to present some facts which may serve as a starting point for further consideration and discussion by others.

Under the head "Dental Pathology," F. Maury, specified more than fifty forms of disease, only a brief notice of each, instead of report, would comprehend a treatise.

Caries or decay of teeth, is that form of disease which, of all others, most frequently comes under the notice of the dental practicioner, and for the preventing or arresting of which his services are constantly in requisition.

Caries is the gradual distruction of a part, or the whole of the dental structure. It manifests itself by an opaque or brown appearance (in a few instances by a chalky whiteness) on the exterior of the teeth, and occasions a softening and wasting, first of the enamel and then of the dentine; and unless arrested by timely and judicious surgical treatment results in total loss of the dental structure.

That there is in the teeth of some individuals a hereditary and constitutional tendency to decay is unquestionably true, and may be clearly demonstrated. But the causes of deçay are so diversified, that in many cases it is extremely difficult to furnish reliable diagnosis. The surgical treatment is familiar to every dental practioner. We all recognize four essentials: The perfect and thorough removal of all carious matter; properly shaping the cavity so as to retain the material employed for filling; drying and keeping dry the cavity during the entire process of filling; to fill perfectly so as to exlude air and moisture, either with gold or whatever material the circumstances may seem to demand.

When the condition of a tooth is such as to admit the use of pure gold in some of the many forms in which it is now prepared for filling teeth, it is unquestionably the best material of all others for that purpose. Other material, such as Albane stopping, Wood's plastic metal, os-artificial, Guillois' cement, and various preparations of amalgam, etc., are now being used, and though not equal to gold in point of purity and durability, yet it is better to employ material of an ferior quality than not to operate, than to do nothing at all. The material is not of so much consequence as the manner of manipulating it.

Preventive is better than cure; and it should be the primary object and constant study of those who engage in the healing art to *guard against and prevent* disease as well as to restore to health.

Then it is an important question, How may caries, exostosis, spinal, ventosa, necrosis, and other diseases to which the teeth and buccal cavity are subjected, be prevented?

Whatevcr tends to strengthen and invigorate the entire physical system, contributes to the preservation and healthfulness of the dental organism.

In this connection may be mentioned a proper attention to *diet.* A sufficient amount of *exercise*, pure *air* and *clealiness*, all contribute in their measure to the preservation of the general health, and as a consequence to the preservation and healthfulness of the teeth.

As to diet it may be either animal or vegetable. Man's structure and appetite indicate that he was formed for both.

Judgment, however, is requisite in adjusting the due proportions of each. Improper diet impairs the mind as well as the body. How this effect is produced has been and is ever likely to remain one of nature's secrets. But that there is a reciprocal influence between the mental and corporial is an established fact.

Exercise next to food is necessary to the preservation of health. We see that those who are constantly and actively employed are not only the most healthy, but the most happy. It is absolutely impossible to enjoy health when perspiration is obstructed, and this cannot be carried on when exercise is neglected. An active life is the great guardian of virtue, as well as the best preventive of disease.

Pure air is not only a means of curing disease but of preserving the system in a healthy state. In its restorative influence, it is often more efficacious than medicine.

Many persons who have a careful regard as to what they eat and drink, pay but little attention as to the purity or impurity of the air they take into their lungs; yet the inhaling of impure air often proves more deliterious to health than vitiated food.

Cleanliness may be regarded as a virtue, a moral excellence and is so agreeable to nature and so essential to health, and water is provided in such abundance, that a want of cleanliness is inexcusable.

We discover that the various functions of the body are intimately connected, and dependent one upon another, and when any one member becomes diseased, the chain of morbid action may run through the entire system, so that in treating any one it is necessary to have regard to its bearing and influence on all the others; and it is essential that the practicing dentist should study all the collateral branches or sciences, that can in any way directly or indirectly bear upon his own

